# Exploitation of the Nutraceutical Potential of the Infesting Seaweed *Chaetomorpha linum* as a Yellow Mealworms’ Feed: Focus on Nutrients and Antioxidant Activity

**DOI:** 10.3390/foods14020325

**Published:** 2025-01-20

**Authors:** Annalaura Brai, Edoardo Brogi, Franca Tarchi, Federica Poggialini, Chiara Vagaggini, Sauro Simoni, Valeria Francardi, Elena Dreassi

**Affiliations:** 1 Department of Biotechnology, Chemistry and Pharmacy, University of Siena, Via A. Moro, 53100 Siena, Italy; edoardo.brogi@student.unisi.it (E.B.); federic.poggialini@unisi.it (F.P.); chiara.vagaggini@student.unisi.it (C.V.); elena.dreassi@unisi.it (E.D.); 2 Research Centre for Plant Protection and Certification (CREA-DC), Via di Lanciola 12/A, 50125 Firenze, Italy; franca.tarchi@crea.gov.it (F.T.); sauro.simoni@crea.gov.it (S.S.); valeria.francardi@crea.gov.it (V.F.)

**Keywords:** edible insects, *Tenebrio molitor*, waste reduction, infesting seaweeds, circular economy, proteins, gastrointestinal digestion

## Abstract

According to the Sustainable Blue Economy Communication, the Farm to Fork Strategy, and the European Green Deal, novel business models supporting the use of resources from oceans and seas are of primary importance. Interestingly, several infesting seaweeds are consumed as foods in Asia and are fundamental ingredients in several traditional dishes. Herein, according to the circular economy approach, we investigate the use of *Chaetomorpha linum* (CL) as a *Tenebrio molitor* larvae feed in different percentages: 20%, 50%, and 100%, respectively. We analyzed the effects of CL on the survival and biometric parameters of larvae. The survival rate remained comparable to the control, while the mean weight was inversely proportional to the CL%. The nutrient composition was substantially different from the control, showing increased protein and reduced fat content. Analysis of the secondary metabolites revealed a better fatty acid composition, rich in PUFA, with lipid nutritional indexes comparable to seaweeds and crabs. The simulated gastrointestinal digestion highlights the increased content of essential amino acids, and a two times higher antioxidant activity with respect to the control. Taken together, our results confirm that CL can be used as a TML supplement, with beneficial effects in protein content and fat quality.

## 1. Introduction

Only 2% of human food comes from the seas and oceans, although they make up more than 70% of the planet’s surface [1,2]. As a result, the Sustainable Blue Economy Communication, the Farm to Fork Strategy, and the European Green Deal all strongly support using algae as a food and feed source, and suggest creating novel business models to boost the production of algae [2,3]. Algae are a low-carbon footprint food that also contains high levels of proteins, fiber, and nutraceutical compounds [4].

Algae range significantly in size and form, from unicellular microscopic algae called microalgae to giant multicellular algae called seaweeds (SWs). There are approximately 25,000–30,000 species of algae. Rhodophytas, or red algae; Chlorophytas, or green algae, both belonging to the Plantae kingdom; and Ochrophytas, or brown seaweed, are the three main groups in which SWs are divided based on the pigment that sets them apart [5]. Because they can swiftly adapt to new environmental conditions, such as significant temperature variation, alteration of salinity, UV-vis irradiation, and nutrient availability, certain algae can survive in extreme environments. Algae are valued for their rapid growth, simplicity of cultivation, and capacity to control the generation of bioactive components by altering the growing environment. They thereby produce a variety of species-specific secondary physiologically active metabolites [6]. We recently evaluated the nutritional and nutraceutical profile of two seaweeds that are invading Orbetello Lagoon, in Tuscany: *Chaetomorpha linum* (CL) and *Valonia aegagrophila* (VA). Both algae are used as ingredients in Asian preparations but are disposed of as waste in Italy. CL has the most promising nutraceutical value [7]. CL is a complete source of digestible proteins; it contains all the essential amino acids, has a low quantity of high-quality fat, and is rich in unsaturated fatty acids [7]. CL is particularly rich in antioxidant compounds and has an ACE inhibitory activity that represents an important nutraceutical added value.

In 2021, *Tenebrio molitor* larvae (TML) have been approved for human consumption by the European Food Safety Authority (EFSA). These larvae are a complete source of digestible proteins, and their fat is mainly composed of mono and polyunsaturated fatty acids [8,9]. TML have high adaptability and the capability to convert low-quality feed into high-quality nutrients [10]. In this context, agri-food by-products have been efficiently used as a feed supplement, increasing TML nutraceutical value. Tomato peels and seeds, as well as other agricultural wastes like fallen leaves, increased the antioxidants in TML [11]. The mean weight and antioxidant activity of TML were increased by the addition of essential oils to post-distillation residues of rice bran, corn cob, and potato peels [12]. Conversely, TML were poisoned or repulsed by the essential oils of oregano, thyme, garlic, and caraway due to the presence of numerous secondary plant metabolites, particularly terpenes, terpenoids, and certain aldehydes [13,14,15]. Due to their greater nutrient content, dried potatoes might accelerate development either by themselves or in combination with egg whites [16]. Similarly, distillery by-products’ high protein content raised TML’s mean weight [17]. Therefore, brewer’s spent grains, bread and cookie leftovers, and combinations of brewer’s spent grain or bread and cookie were used as feed [18,19]. This showed that protein content is crucial for accelerating TML growth, but it also highlighted the importance of maintaining a proper balance for lipids, especially PUFA. According to these findings, larvae fed nutrient-poor substrates have a higher fat proportion but a lower protein content overall [20]. On the contrary, diets rich in proteins result in faster development and greater larval survival.

According to a recently published study [7], CL is rich in proteins and antioxidant compounds, which can increase the development and the shelf life of TML. To valorize the algal waste and upcycle the high content of proteins and antioxidants already found in CL [7], we evaluate herein the possible use of CL as a feed for TML. TML were fed with CL in different proportions, and the analysis of survival rate and biometric data were performed at different time points. TML were processed, and macronutrients were quantified. The analysis of secondary metabolites was then performed, and fatty acids and sugars were analyzed. Finally, TML extracts were subjected to gastrointestinal simulated digestion, and essential amino acids, and antioxidant activity were determined.

## 2. Materials and Methods

### 2.1. Materials

Solvents are reactants, which were provided by Sigma-Aldrich S.r.l. (Milan, Italy). *Tenebrio molitor* larvae (TML) were reared at the Research Centre for Plant Protection and Certification, (CREA-DC Florence, Italy). TML were kept in a semi-dark environment at 27 ± 1 °C, and relative humidity comprised between 40 and 50% [21]. *Chaetomorpha linum* (CL) was a kind gift of the “Orbetello pesca lagunare” association (Orbetello, Grosseto, Italy).

### 2.2. Design of the Experiment

The control group (CTRL) was fed with brewer’s yeast (0.5%), wheat flour (49.75%), and oats (49.75%), as previously published [22]. CL was sun-dried, and water content was decreased to 10% *w*/*w* via drying under a vacuum at 20° C for 48 h. Dried CL was finely crushed with a blender TSM6A013B, (Bosh, Gerlingen-Schillerhoehe, Germany), and filtered with a 500 µm Endecotts test sieve (Endecotts Ltd., London, UK). The control diet was replaced with CL in different proportions, ranging from 20 to 100%.

**Figure 1:**
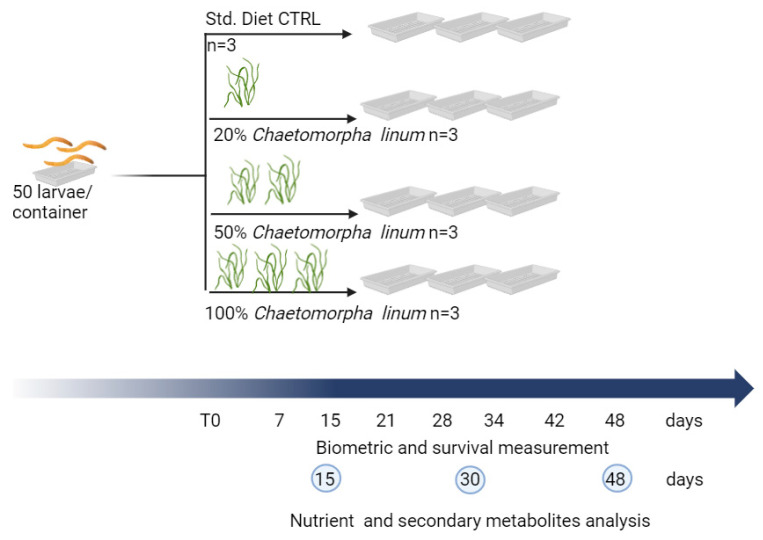
Design of the trial. Three plastic trays containing 50 larvae/each, were fed with a standard diet (CTRL); three containers were fed with a diet containing 20% *Chaetomorpha linum* (CL20%); three containers were fed with a diet containing 50% *Chaetomorpha linum* (CL50%); and three containers were fed with a diet containing 100% *Chaetomorpha linum* (CL100%). At various time points (7, 15, 21, 28, 34, 42, and 48 days) survival and biometrical measurements were made. Nutrients and secondary metabolites were analyzed after 15, 30, and 48 days.

The 30-day-old larvae, (length comprised between 15 and 20 mm) were used for the experiment. In total, 50 TML per container were randomly divided into four experimental groups, that received the Standard Diet (CTRL) or the alternative diets (CL20%, CL50%, and CL100%) (Figure 1). Each experiment was performed in triplicate. At 7, 15, 21, 28, 34, 42, and 48, survival rates, and biometric data were made. The larvae were freeze-dried with an Edward MOD. freeze-dryer (Edward and Co., Ltd., London, UK) and kept at −20 °C prior to analysis.

### 2.3. Extraction of Polar Metabolites

TML were extracted as previously reported [21]. In detail, 10 mL of a MeOH/H_2_O (1:1 ratio) mixture was used to extract 1 g of dried TML. The sample was homogenized with an IKA Labortechnik T25 basic (IKA Werke GmbH and Co., Staufen, Germany). After centrifugation for 5 min, the supernatant and pellet were separated, and the solvent was recovered. The procedure was repeated two times, and the solvent was evaporated under a N_2_ flow. The extracts were frozen at −20 °C prior to analysis.

### 2.4. Proximate Composition

The water content was determined starting from 1 g of each sample. The sample was weighed and dried over 14–16 h at 105 °C. The initial weight was subtracted from the final weight to calculate the water content. The same procedure has been adopted to calculate the moisture content starting from already-dried samples.

Starting from 1.0 g of dried material, the Kjeldahl method was used to determine the nitrogen content. A coefficient of 4.76 was used to determine the crude protein content [23].

The following conversion factors were used to determine energy values: 3.87 kcal/g for carbohydrates, 9.02 kcal/g for fats, and 4.27 kcal/g for proteins.

Fat was determined using the Folch method [24]. TML (1 g) were homogenized for 5 min using an IKA Labortechnik T25 basic (IKA WERKE GmbH and Co., Staufen, Germany) and extracted with CHCl_3_. After washing with 10 mL of a KCl 0.7% solution, the organic layer was filtered and washed twice with the “upper phase” (prepared from CHCl_3_/CH_3_OH/H_2_O (53:27:20 *v*/*v*) mixture), and dried. Before being used for FA analysis, the extract was weighed to determine its fat content.

### 2.5. Gastrointestinal Digestion

The in vitro determination of gastrointestinal digestion (GID) was originally published in [25] with a few adjustments. In total, 500 mg of dried TML were homogenized using an IKA Labortechnik T25 basic (IKA Werke GmbH and Co., Staufen, Germany) for 5 min in 5 mL of a TRIS/HCl buffer, 50 mM, and pH 7.4. Then, pepsin (enzyme/substrate 1:250 *w*/*w*) was added, and HCl 1M was added to bring the pH down to 2. The mixture was kept at 37 °C for two hours. The impact of gastric digestion (GD) has been assessed using the extract.

After raising the pH to 6.5 with 1 M NaOH, a 1:1 combination of trypsin and α-chymotrypsin (enzyme/substrate 1:250 *w*/*w*) was added. The mixture was kept at 37 °C for two hours. Following a 10 min centrifugation at 74,689× *g*, the supernatant was collected and filtered using 3 kDa filters (Amicon Ultra Milli-pore Sigma, Burlington, MA, USA). The samples were then stored at −20 °C.

### 2.6. NMR Analysis

In total, 1.0 mL of a 400 mM phosphate buffer/D_2_O (pH 7) containing 1 mM 3-(trimethylsilyl)propionic acid sodium salt (TSP) as a standard was used to dissolve the digested extracts. Before analysis, the samples were filtered. A Bruker Advance DPX 400 MHz spectrometer (Bruker Biospin, Billerica, MA, USA) was used to record monodimensional ^1^H NMR at T = 298 K. The ^1^H NMR were obtained using a single pulse experiment that included 16 scans, 4 dummy scans, a 3 s relaxation delay, and a 90° excitation pulse and the Bruker zgpr sequence. Chenomx NMR suite v 11 (Chenomx Inc., Edmonton, AB, Canada) and already published data [10,18] were used to assign ^1^H NMR spectra and identify secondary metabolites.

### 2.7. Fatty Acids

The fatty acids methyl esters (FAMEs) were produced and analyzed as previously published [21,26]. After a one-hour reaction at 100 °C with 1 mL of BF_3_ methanolic solution, the lipophilic extract (5 mg) was cooled, and the FAME was extracted using 1 mL of n-hexane for GC analysis.

According to earlier reports, a GC-FID (Clarus 500 GC, Perkin Elmer Norwalk CT) was used for the GC-FID analyses [21,26].

The Perkin Elmer TotalChrom Navigator software version 6.3.1 was used to analyze the data. Different FAs were identified by comparing their retention times to the available standards and data already reported [21,26].

### 2.8. Lipid Quality Indices Determination

Indices of thrombogenicity (IT), atherogenicity (IA), and Hypocholesterolemic/Hypercholesterolemic (HH) ratio were calculated based on FA composition as previously described [27] using the following formulas:

(1) $$IT=\frac{\left(C14:0)+(C16:0)+(C18:0\right)}{\left(0.5\times MUFA\right)+\left(0.5\times Cn6\right)+\left(3\times Cn3\right)+\left(n3\times Cn6-1\right)}$$

(2) $$IA=\frac{\left(C12:0)+(4\times C14:0)+(C16:0\right)}{PUFA(Cn6\;and\;Cn3)+MUFA)}$$

(3) $$HH=\frac{cis-C18:1+\Sigma PUFA}{C12:0+C14:0+C16:0}$$

### 2.9. Amino Acid Determination

Amino acids were quantified after gastrointestinal hydrolysis. Utilizing the Fmoc pre-derivatization process and the previously documented chromatographic conditions [25], AAs were quantified by HPLC using an Agilent 1260 Infinity instrument (Agilent Technologies, Palo Alto, CA, USA) [25]. Acetonitrile was used as eluent B and 50 mM acetate buffer (pH 4.2) as eluent A in an LC-18 column (Gemini NxC18, 4.6 × 250 mm, 5 μm) to achieve separation. At 25 °C, the column operated at 1.0 mL/min. The amino acids were separated using the following linear gradient elution conditions (min/A%): 0/72, 3/72, 27/55, 32/0, 37/0, 39/72, and 47/72 and their concentration determined by interpolation from a standard curve with the opportune standards.

### 2.10. Determination of the Antioxidant Activity

The antioxidant activity of insect extracts after simulated oral, gastric, and gastrointestinal digestion was determined using an ABTS assay. Trolox has been used as a reference compound, and the antioxidant activity is expressed as TEAC (µmol of Trolox equivalent per gram of dry matter). In detail, 0.1 mL of the opportune extract was added to 1.5 mL of an ABTS solution and 1.4 mL of EtOH. The mixture was vortexed, and after 30 min, the absorbance was recorded at 751 nm (UV/Visible Lambda 2 spectrophotometer, Perkin-Elmer, Norwalk, CT, USA).

### 2.11. Statistical Analysis

The results were presented as mean ± SD of three independent analysis. GraphPad Prism 8.2 was used for statistical analysis (GraphPad Software, La Jolla, CA, USA). The Brown–Forsythe and Shapiro–Wilk tests were used to confirm the homoscedasticity and normality of the data.

The Mantel–Cox test was used to examine the Kaplan–Meier graph, and results with *p* ≤ 0.05 were deemed significant.

The groups’ proximate composition, fatty acids, and polar metabolites were compared using a one-way analysis of variance (ANOVA). ** *p* ≤ 0.01 * *p* ≤ 0.05 vs. CTRL *** *p* ≤ 0.001 vs. CTRL were used to indicate significant differences. The mean increase in body weight and the amount of fat over time were examined using a two-way ANOVA with varied CL% and rearing durations (15, 30, and 48 days) as fixed factors. Two-way ANOVA was used to consider differences between antioxidant activities after simulated gastrointestinal digestion, considering digestion steps and diets as fixed factors.

## 3. Results and Discussion

### 3.1. Analysis of Survival Rate and Body Weight

**Figure 2:**
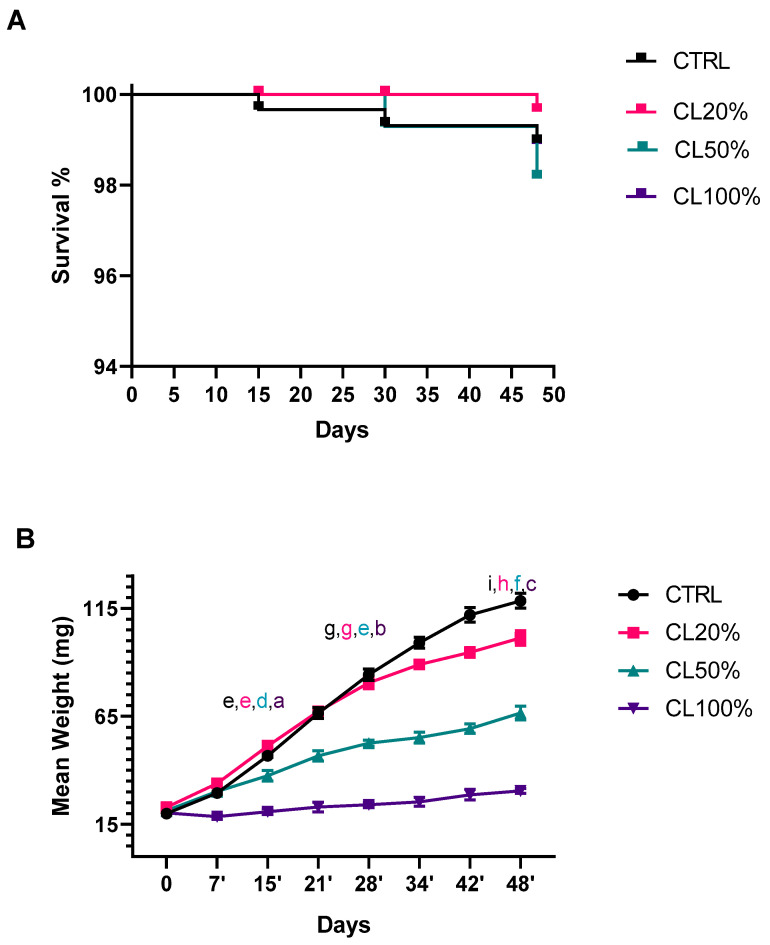
(**A**) Kaplan–Meier curve of *Tenebrio molitor* larvae fed on a standard diet (CTRL) or a standard diet supplemented with *Chaetomorpha linum* (CL). Larvae supplemented with 20% (*w*/*w*) CL, 50% (*w*/*w*) CL (CL50%), or 100% (*w*/*w*) CL (CL100%). Curve comparison has been analyzed with the Mantel–Cox test (χ^2^ = 2.677, df = 3, and *p*-value = 0.4441). (**B**) Mean body weight over time. Statistical analysis was performed with two-way ANOVA with different diets and time of rearing (15, 28, and 48 days) as fixed factors and Tukey’s posthoc test. a, b, c, d, e, f, g, h and i values without a common superscript are significantly different (*p* < 0.05).

As reported in the Kaplan–Meier curve (Figure 2A), the survival rate was not affected by the administration of CL in different proportions, and was statistically comparable to the cereal-based diet used as a control. The quantity of CL were selected based on the previous analysis performed on CL and on the use of agrifood byproducts on TML [10,11,17,21]. On the one hand, this result is not surprising, because seaweeds, such as *Ulva*, *Saccharina*, and *Laminaria* species have the potential to furnish the protein and energy needs of livestock, according to in vivo studies on ruminants, pigs, poultry, and rabbits [22,23,24,25,26,27,28,29,30,31,32,33,34]. Other seaweeds contain a variety of bioactive compounds that could be used as prebiotics to improve the health and productivity of both monogastric and ruminant livestock [23,35,36]. On the other hand, *Ulva* species, in particular *U. fasciata* and *U. lactuca*, reduced both the fecundity and longevity of the red cotton bug *Dysdercus cingulatus* [37]. The brown alga *S. latifolium* was recently proposed as an insecticide, due to its important toxicity against *Arcophaga bullata*, *Tribolium castaneum*, *Musca domestica*, and *Solenopsis invicta* larvae [38].

It has been previously demonstrated that the red alga *Ascophyllum nodosum* can be administered as a feed to black soldier fly larvae, without affecting vitality [39]. CL is rich in protein (29% of DW) and poorer in fats (less than 2%), a nutrient composition that can speed up the larvae’s growth [7]. In fact, our standard diet has a high content of carbs (70%) but a lower protein content (14.5%) [11]. Considering the mean weight over time (Figure 2B), we found that the administration of CL20% did not affect the mean weight in the first 28 days, while slightly decreasing the value from 28 to 45 days. The mean weight is inversely proportional to the CL percentage, reaching the worst value in CL100% groups, which showed an altered development, probably due to the low quantity of accessible carbohydrates, necessary for development and the mutation to the pupal stage. Considering the mean weight, only CL20% is suitable for industrial mass rearing. Taken together, these findings underline that toxic effects are species-specific, and additional studies are worthy of investigation.

### 3.2. Analysis of Proximate Composition

The larvae’s proximate composition underlined important modifications (Table 1). The protein content increased proportionally to the CL%, reaching comparable values in the CL50% and CL100% groups. A conversion factor of 4.76 was used to discriminate the chitin contribution [40]. A similar trend has been observed using protein-rich by-products, in particular mozzarella whey, whey permeates, *Moringa oleifera* leaves, and chestnut shells [10,41,42]. The fat was inversely proportional to the CL percentage. Previous animal studies demonstrated that macroalgae prevented the accumulation of fat and other consequences of obesity, including fatty liver, insulin resistance, and dyslipidemia. In white adipose tissue, macroalgae can limit the availability of fatty acids for triglyceride synthesis by reducing de novo lipogenesis. The ability of macroalgae to reduce body fat may be attributed to improved thermogenic capability and fatty acid oxidation, as well as a change toward a more wholesome composition of gut microbiota [43]. Even if further studies are necessary to elucidate the mechanism involved in weight and fat reduction, the CL% strongly correlates with both mean weight and fat reduction.

The carbs increased proportionally to the CL%, reaching the highest value in CL100%. This is not surprising due to the high content of carbohydrates already found in CL [44]. The NMR analysis underlined glucose as the most abundant monosaccharide and sucrose among disaccharides (Supplementary Information Figure S1). The presence of glucose as the main monosaccharide was similar to the control, while in previous experiments with substrates like wheat bran or oats supplemented with chestnuts or milk industry by-products, the main monosaccharide was rhamnose [10,42]. Due to the insolubility of chitin at pH 7.4, the chitin content was not investigated in the study.

When compared to other meats, TML’s protein level is lower than that of beef and chicken but in line with or higher than that of lamb and pig [45]. On the other hand, fat content is lower than that of lamb and pig but higher than that of beef and chicken. Based on the basal metabolic rate, the FAO and WHO advise a 75 kg man to consume between 2550 and 4000 kcal per day to meet his energy needs [46]. Athletes need to consume a lot of energy and a different amount of nutrients; accordingly, protein intake can reach up to 2.2 g/kg of body weight, while carbohydrates should be raised to 12 g/kg of body weight. Regarding fat, the suggested amount is between 20 and 35 percent of total fat intake [46,47,48]. In light of those values, TML can meet the needs of athletes and regular people for fat and protein, while carbohydrates fall outside the ideal range as typically observed for pork, lamb, or chicken meats.

### 3.3. Analysis of Fat

**Figure 3:**
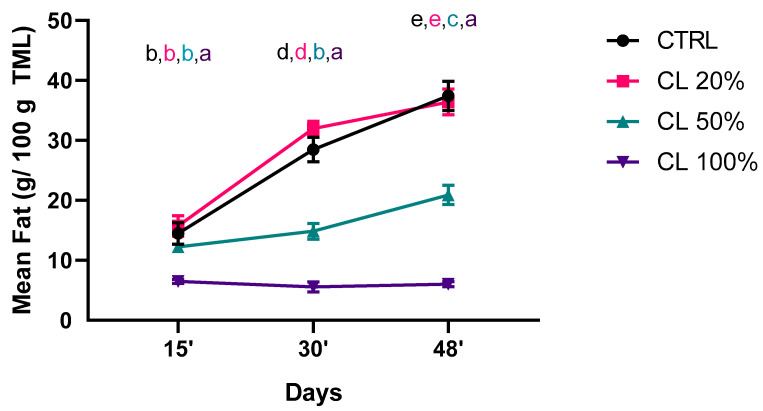
Fat (g of fat/100 g of TML) quantification of *Tenebrio molitor* larvae fed on a standard diet (CTRL) or a standard diet supplemented with *Chaetomorpha linum* (CL) 20% (*w*/*w*) CL, 50% (*w*/*w*) CL (CL50%), or 100% (*w*/*w*) CL (CL100%) over time. The results represent the mean ± SD of three experiments. Statistical analysis was performed with two-way ANOVA with different diets and time of rearing (15, 30, and 45 days) as fixed factors and Tukey’s post hoc test. a, b, c, d and e values without a common superscript are significantly different (*p* < 0.05).

As discussed in Section 2.3, the fat was inversely proportional to the CL percentage. Using time and diet as fixed factors, the mean fat was calculated throughout the experiment and examined using two-way ANOVA (Figure 3). The effect of time (df = 3, F = 412.4, and *p* < 0.0001), and diets (df = 2, F = 220.4, and *p* < 0.0001) were both statistically significant. After 15 days (Figure 3), a CL percentage comprised between 20 and 50% led to a mean fat content statistically comparable to the standard diet. Noteworthy, the total replacement with CL reduced the mean fat. After 30 days only the lowest percentage of CL maintained the mean fat comparable with CTRL, while we observed a reduction for both CL50% and CL100% experimental groups, which was maintained after 48 days. Interestingly, the CL100% group had a mean fat statistically comparable from 15 to 45 days, underlying the potential effect of pure CL100% in metabolism. Fat reduction follows the same trend observed for mean weight, even if, after 48 days, an additional fat reduction has been observed.

As shown in Table 2, TML retained their usual fatty acid composition, abundant in monounsaturated fatty acids (MUFAs) and polyunsaturated fatty acids (PUFAs). In CL20% and CL50% groups, short chain fatty acids (SFA) were statistically reduced to CTRL, due to a significant reduction in both C16:0 and C18:0. MUFA remained comparable or slightly below the CTRL; among MUFA, we observed an increased value of palmitoleic acid, abundant in CL [7,49]. We also observed an increased value of PUFA directly proportional to CL content, which was mainly due to an increased content of linolenic acid. These values are not surprising, because seaweeds and CL are particularly rich in PUFA, in particular linoleic acid. In addition, previous studies demonstrated that the alga *Ascophyllum nodosum* increases PUFA in black soldier fly larvae [39]. Even if present in CL, EPA and DHA were not detected in TML extracts [7,49].

An elevated risk of cardiovascular disease may be linked to SFA quality. While C18:0 is thought to be thrombogenic but neutral in terms of atherogenicity, C14:0 and C16:0 are two of the most atherogenic SFAs. The nutritional indices linked to elevated risks of cardiovascular disease were computed. The ratio of total SFAs to total unsaturated fatty acids (UFAs) is known as the atherogenicity index (IA), which was developed by Southgate [50]. The computed values, which ranged from 0.35 to 0.44, were ameliorated, with values below CTRL. While the IAs of quick foods such beef burgers and Margherita pizza are higher, and comprised between 0.99 and 1.99, respectively, the TML values are comparable to those reported for crops, chicken, and brown and green seaweeds [51,52,53]. The ratio of saturated thrombogenic fatty acids to unsaturated anti-thrombogenic fatty acids is known as the thrombogenicity index, and it indicates the risk of clots developing in the arteries. IT were significantly ameliorated with CL addition. IT values are lower than meats like lamb and rabbit (1.2 and 1.1, respectively), or processed meals like Bologna sausages (1.55), beef burgers (1.40), and Margherita pizza (2.42) but it is comparable to the range seen for brown seaweeds and chicken [51,53,54,55].

The hypocholesterolemic/hypercholesterolemic ratio (HH) was calculated. As shown in Table 2 the values were both increased, reaching values comparable to rock crabs [56], significantly higher than lamb, cattle and chicken meats [53]. CL is rich in MUFA and PUFA and has high-quality fat [7,49]. The obtained results were not surprising because as previously demonstrated, TML can accumulate lipophilic substances from feeds and biosynthesize MUFA and PUFA, further increasing the quality of fat [10,57,58,59].

### 3.4. Analysis of Polar Secondary Metabolites

TML were subjected to simulated gastrointestinal digestion, to evaluate polar secondary metabolites generated. TML bolus was first incubated with pepsin at pH 2 for two hours to mimic gastric fluids. Subsequently, the digested samples were incubated with trypsin and chymotrypsin at pH 6.5 to mimic duodenal digestion.

**Figure 4:**
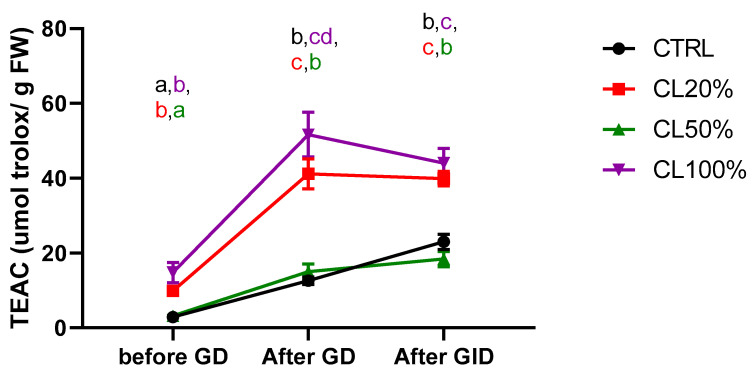
Trolox equivalent antioxidant capacity (TEAC) analysis before gastric digestion (GD), after gastric digestion (GD), and after gastrointestinal digestion (GID). *Tenebrio molitor* larvae were fed on a standard diet (CTRL) or a standard diet supplemented with *Chaetomorpha linum* (CL). The results represent the mean ± SD of three experiments. Larvae supplemented with 20% (*w*/*w*) CL, 50% (*w*/*w*) CL (CL50%), or 100% (*w*/*w*) CL (CL100%). Statistical analysis was performed with two-way ANOVA with different diets and digestion steps as fixed factors and Tukey’s post hoc test. a, b, c and d values without a common superscript are significantly different (*p* < 0.05).

Among the secondary metabolites, antioxidants are of primary importance and can shield the body from oxidative stress-related damage. According to previous studies, oxidative stress is triggered by free radicals and is a key contributor to the pathophysiology of conditions like cancer, alcoholic liver cirrhosis, and atherosclerosis [60]. Indeed, both lipophilic and hydrophilic antioxidants are abundant in CL. Among hydrophilic compounds, CL is rich in phlorotannins, polyphenols, flavonoids, and proanthocyanidins [7,61]. Among lipophilic antioxidants, CL is rich in carotenoids xanthophylls, and chlorophylls [7,56]. As we can observe in Figure 4, the Trolox equivalent antioxidant capacity (TEAC) increased after gastric digestion (GD) and maintained values statistically comparable after completing intestinal digestion. Interestingly, the higher TEAC were found in CL100% group, followed by CL20% and CL50%.

Samples from GID were also evaluated for their content of essential amino acids. CL groups had a higher number of amino acids, which reflects the increased protein content. However, even if characterized by high protein digestibility, the amino acids did not reflect the total aminoacidic content because of the lack of hydrolysis in strong acids. According to previously published data, aromatic amino acids were the most prevalent amino acids [10,18,49]. As shown in Table 3, TML have all the essential amino acids and a balanced amino acid composition. CTRL possesses three limiting amino acids, Thr, Cys, and Leu, while above the acceptable score for all other necessary amino acids, in accordance with FAO and WHO recommended levels. Lys concentrations are reduced in CL20% and CL50%. Thanks to the increased amino acid content, TML groups satisfy the recommended total value of essential amino acids. Considering the suggested essential aminoacidic requirements, a man of 70 kg body weight needs to consume daily 55 g of dried TML.

## 4. Conclusions

In conclusion, our work shows that the invasive species CL can be successfully upcycled as TML feed up to 20% of the total feed weight. Higher concentrations were detrimental to the mean weight of TML, an essential parameter in mass rearing. The nutrient composition was substantially different from the control, showing an elevated protein value, while the mean weight and survival rate stayed the same. Analysis of the secondary metabolites revealed a better fatty acid composition, with lipid nutritional indexes comparable to seaweeds and crabs. We observed increased concentrations of PUFA, as well as reduced IA and IT, and increased HH. All these values demonstrated a statistically significant variation in the fat quality, which was ameliorated with respect to CTRL. Notably, these values are related to a lower risk for cardiovascular diseases, and represent an added value of TML. The simulated gastrointestinal digestion highlights an increased content of essential amino acids, which satisfy the values suggested by FAO/WHO/UNU. Finally, we observed a two-time higher antioxidant activity, which can increase shelf life and reduce ROS-related stress. Future efforts will be dedicated to the search of by-products combinations able to maintain the high protein quantity and the quality of fat obtained in this study but also increase mean weight and egg production.

## Figures and Tables

**Table 1:** Analysis of the proximate composition of *Tenebrio molitor* larvae reared on standard and supplemented feeds after 48 days of rearing ^#^.

% Composition	CTRL ± SD	CL20% ± SD	CL50% ± SD	CL100% ± SD
Proteins (% DW)	36.20 ± 1.73 ^a^	40.13 ± 1.66 ^a^	44.16 ± 1.96 ^b^	43.16 ± 1.91 ^b^
Fat (% DW)	37.42 ± 1.40 ^c^	36.41 ± 2.14 ^c^	20.91 ± 1.61 ^b^	6.02 ± 0.52 ^a^
Carbohydrates (% DW)	6.56 ± 0.55 ^a^	4.97 ± 0.62 ^a^	7.65 ± 0.71 ^a^	14.56 ± 0.98 ^b^
Energy (kcal/100 g DW)	517.49 ± 22.53 ^c^	519.01 ± 28.79 ^c^	402.17 ± 25.64 ^b^	295.29 ± 14.70 ^a^

^#^ Results represent the mean ± SD of three experiments. Larvae supplemented with 20% (*w*/*w*) CL (CL20%), 50% (*w*/*w*) CL (CL50%), or 100% (*w*/*w*) CL (CL100%). The different superscript letters of a, b, c, indicate a significant difference among the means in each column; *p* < 0.05 by post hoc Tukey’s test.

**Table 2:** Analysis of FA composition and lipid quality indices of *Tenebrio molitor* larvae reared on standard and supplemented feeds after 48 days of rearing ^#^.

	CTRL		CL20%		CL50%		CL100%	
	Mean	SD	Mean	SD	Mean	SD	Mean	SD
C10:0	0.010	0.000	0.021	0.000	0.020	0.010	0.011	0.000
C12:0	0.495	0.016	0.513	0.002	0.595	0.134	0.968	0.011
C13:0	0.041	0.000	0.036	0.005	0.049	0.001	0.032	0.001
C14:0	5.155	0.010 ^b^	4.948	0.362 ^b^	4.070	0.150 ^b^	2.210	0.074 ^a^
C15:0	0.051	0.000	0.062	0.000	0.175	0.012	0.075	0.001
C16:0	13.348	0.283 ^b^	12.336	0.131 ^a^	12.244	0.143 ^a^	16.792	1.063 ^c^
C17:0	0.056	0.005	0.062	0.000	0.117	0.002	0.128	0.002
C18:0	2.901	0.033 ^a^	2.761	0.229 ^a^	2.931	0.126 ^a^	4.668	0.270 ^b^
C20:0	0.092	0.010	0.113	0.011	0.117	0.011	0.214	0.025
Σ SFA	22.148	0.295 ^b^	20.850	0.731 ^a^	20.317	0.303 ^a^	25.099	1.425 ^c^
C14:1 11	0.347	0.011	0.431	0.033	0.175	0.012	0.075	0.001
C16:1 9	1.851	0.068 ^a^	1.996	0.088 ^a^	2.527	0.145 ^ab^	3.466	0.050 ^b^
C16:1 11	1.178	0.047	1.329	0.053	0.866	0.002	0.476	0.019
C18:1 9	53.225	0.230	53.782	0.800	51.012	0.340	41.632	1.604
C18:1 11	0.000	0.000	0.000	0.000	0.000	0.000	0.000	0.000
C20:1 11	2.239	0.323	1.747	0.706	1.272	0.177	2.058	0.810
Σ MUFA	58.849	0.679 ^c^	58.853	0.130 ^c^	55.677	0.016 ^b^	47.631	0.863 ^a^
C14:2 9,11	0.163	0.000	0.149	0.015	0.127	0.002	0.048	0.005
C16:2 9,11	0.163	0.000	0.159	0.015	0.229	0.012	0.124	0.114
C18:2 9,12	18.646	0.354 ^a^	19.907	0.572 ^b^	23.247	0.297 ^c^	26.093	0.603 ^d^
C18 d3 6 9,12	0.194	0.030	0.231	0.014	0.531	0.021	1.053	0.074
Σ PUFA	19.002	0.384 ^a^	20.297	0.601 ^a^	24.006	0.287 ^b^	27.270	0.562 ^c^
IA	0.443	0.006 ^a^	0.413	0.024 ^a^	0.365	0.009 ^b^	0.355	0.025 ^b^
IT	0.538	0.009 ^b^	0.495	0.023 ^a^	0.464	0.004 ^a^	0.590	0.049 ^b^
HH	3.802	0.047 ^b^	4.166	0.195 ^c^	4.437	0.074 ^c^	3.462	0.304 ^a^

^#^ *Tenebrio molitor* larvae fed on a standard diet (CTRL) or a standard diet supplemented with *Chaetomorpha linum* (CL). over time. Results represent the mean ± SD of three experiments. Larvae supplemented with 20% (*w*/*w*) CL, or 50% (*w*/*w*) CL (CL50%), or 100% (*w*/*w*) CL (CL100%). SFA: short-chain fatty acids; MUFA: monounsaturated fatty acids; PUFA: polyunsaturated fatty acids. Atherogenicity index (IA), thrombogenicity index (IT), and hypocholesterolemic/hypercholesterolemic ratio (HH). a, b, c, d Different superscript letters indicate a significant difference among the means in each column, *p* < 0.05 by post hoc Tukey’s test.

**Table 3:** Aminoacidic composition of *Tenebrio molitor* larvae fed on a standard diet (CTRL) or a standard diet supplemented with *Chaetomorpha linum* (CL) at different proportions 20% (*w*/*w*) CL (CL20%), 50% (*w*/*w*) CL (CL50%), or 100% (*w*/*w*) CL (CL100%). Data are compared with FAO/WHO/UNU suggested amino acid requirements in mg/g of protein.

AA	CTRL	CL20%	CL50%	CL100%	FAO/WHO/UNU Reference Protein
His	25.72 ± 1.68	46.20 ± 2.79	48.73 ± 1.54	65.02 ± 1.25	19
Thr	32.14 ± 1.00	36.79 ± 1.16	42.98 ± 1.17	49.55 ± 0.99	34
Val	38.65 ± 1.49	30.21 ± 1.73	30.36 ± 2.23	18.90 ± 1.38	35
Met + Cys	12.64 ± 1.17	14.25 ± 1.39	11.76 ± 1.53	8.75 ± 1.01	25
Lys	59.88 ± 2.21	52.50 ± 1.52	30.07 ± 1.89	79.17 ± 2.63	58
Ile	30.65 ± 2.69	33.24 ± 1.39	31.05 ± 1.81	27.05 ± 1.97	28
Leu	51.24 ± 0.59	56.35 ± 2.22	52.17 ± 1.75	43.87 ± 2.14	66
Phe + Tyr	90.86 ± 4.42	86.39 ± 3.63	59.21 ± 4.31	70.72 ± 2.56	63
Total EAA	341.78 ± 15.25	355.93 ± 15.82	306.33 ± 16.24	363.03 ± 13.93	328

## Data Availability

The original contributions presented in the study are included in the article/Supplementary Material, further inquiries can be directed to the corresponding author.

## Supplementary Materials

The following supporting information can be downloaded at: https://www.mdpi.com/article/10.3390/foods14020325/s1, Figure S1: 1HNMR analysis of *Tenebrio molitor* extracts ^#^.
