# Supplementary material for: Exploitation of the Nutraceutical Potential of the Infesting Seaweed Chaetomorpha linum as a Yellow Mealworms’ Feed: Focus on Nutrients and Antioxidant Activity
Source: Foods. 2025 Jan 20;14(2):325. doi: 10.3390/foods14020325 (PMC11764564; doi:10.3390/foods14020325)
Supplement: Supplementary file 1 [file foods-14-00325-s001.zip › foods-3381299-supplementary.pdf]

Exploitation of the nutraceutical potential of the infesting seaweed *Chaetomorpha linum* as a yellow mealworms' feed: focus on nutrients and antioxidant activity.

Annalaura Brai[1*], Edoardo Brogi[1], Franca Tarchi[2], Federica Poggialini[1], Chiara Vagaggini[1], Sauro Simoni[2], Valeria Francardi[2] and Elena Dreassi[1]

1 Department of Biotechnology, Chemistry and Pharmacy, University of Siena, via A. Moro - 53100 - Siena, Italy

2 Research Centre for Plant Protection and Certification (CREA-DC), via di Lanciola 12/A, 50125 Firenze, Italy

* **Correspondence:** Dr. Annalaura Brai, Department of Biotechnology, Chemistry and Pharmacy, University of Siena, via A. Moro - 53100 - Siena, Italy E-mail: annalaura.brai@unisi.it**.**

Keywords: edible insects; *Tenebrio molitor*; waste reduction; infesting seaweeds; circular economy; proteins; gastrointestinal digestion

Contents

Figure S1. ^{1}HNMR analysis of *Tenebrio molitor* extracts[#]..3

Figure S1. ^{1}HNMR analysis of *Tenebrio molitor* extracts[#]

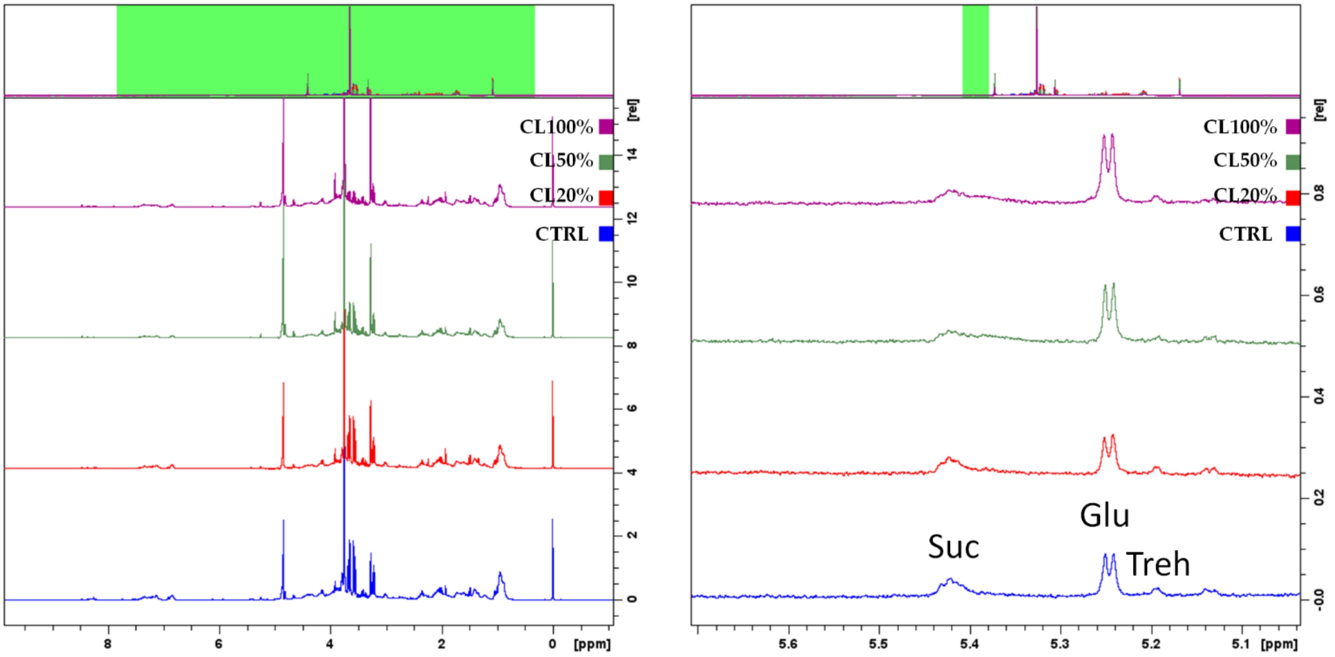

[#] Superimposition of the ^{1}HNMR spectra of the aqueous extracts of *Tenebrio molitor* larvae after gastrointestinal digestion. Standard diet-supplemented larvae (CTRL, blue) fed on a standard diet (CTRL) or standard diet supplemented with *Chaetomorpha linum* (CL). Larvae supplemented with 20% (w/w) CL, or 50% (w/w) CL (CL50%), or 100% (w/w) CL (CL100%). On the left panel is shown the full spectra superimposition, of the right panel the magnification of the sugar region from 5.0 to 5.7 ppm. Suc (sucrose), Glu (glucose), Treh (Trehalose).
